# Novel bioinformatics strategies for prediction of directional sequence changes in influenza virus genomes and for surveillance of potentially hazardous strains

**DOI:** 10.1186/1471-2334-13-386

**Published:** 2013-08-21

**Authors:** Yuki Iwasaki, Takashi Abe, Yoshiko Wada, Kennosuke Wada, Toshimichi Ikemura

**Affiliations:** 1 Department of Bioscience, Nagahama Institute of Bio-Science and Technology, Nagahama-shiShiga-ken, 526-0829, Japan; 2 Department of Information Engineering, Institute of Science and Technology Faculty of Engineering, Niigata University, Niigata-ken 950-2181, Japan

**Keywords:** Influenza virus, Pandemic, Host tropism, H1N1/09, Self-organization map, Oligonucleotide composition, Bioinformatics, Big data, Virus invasion, Zoonotic virus

## Abstract

**Background:**

With the remarkable increase of microbial and viral sequence data obtained from high-throughput DNA sequencers, novel tools are needed for comprehensive analysis of the big sequence data. We have developed “Batch-Learning Self-Organizing Map (BLSOM)” which can characterize very many, even millions of, genomic sequences on one plane. Influenza virus is one of zoonotic viruses and shows clear host tropism. Important issues for bioinformatics studies of influenza viruses are prediction of genomic sequence changes in the near future and surveillance of potentially hazardous strains.

**Methods:**

To characterize sequence changes in influenza virus genomes after invasion into humans from other animal hosts, we applied BLSOMs to analyses of mono-, di-, tri-, and tetranucleotide compositions in all genome sequences of influenza A and B viruses and found clear host-dependent clustering (self-organization) of the sequences.

**Results:**

Viruses isolated from humans and birds differed in mononucleotide composition from each other. In addition, host-dependent oligonucleotide compositions that could not be explained with the host-dependent mononucleotide composition were revealed by oligonucleotide BLSOMs. Retrospective time-dependent directional changes of mono- and oligonucleotide compositions, which were visualized for human strains on BLSOMs, could provide predictive information about sequence changes in newly invaded viruses from other animal hosts (e.g. the swine-derived pandemic H1N1/09).

**Conclusions:**

Basing on the host-dependent oligonucleotide composition, we proposed a strategy for prediction of directional changes of virus sequences and for surveillance of potentially hazardous strains when introduced into human populations from non-human sources. Millions of genomic sequences from infectious microbes and viruses have become available because of their medical and social importance, and BLSOM can characterize the big data and support efficient knowledge discovery.

## Background

While G+C% has long been used as a fundamental parameter for phylogenetic classification of microbial genomes including viral genomes, the G+C% is apparently too simple a parameter to differentiate and characterize a wide variety of genomes. Oligonucleotide composition, however, can be used to distinguish species even with the same G+C%, because the oligonucleotide composition varies significantly among the genomes and is called the “genome signature” [1,2]. Kohonen’s self-organizing map (SOM) is a powerful tool for clustering and visualizing high-dimensional complex data on a two-dimensional plane [3,4]. For oligonucleotide composition handled as high-dimensional data, we modified the conventional SOM to “BLSOM” [5,6], which was suitable for actualizing high-performance parallel-computing and thus for big data such as millions of genomic sequences [7].

On BLSOM for di-, tri- or tetranucleotide composition in genomic sequence fragments (e.g. 10 kb) derived from a wide range of prokaryotic and eukaryotic species, the sequences were found to be clustered (self-organized) primarily according to species on one plane [7,8]. Importantly, BLSOM can visualize the diagnostic oligonucleotides responsible for species-specific clustering, allowing efficient knowledge discovery of the molecular processes of establishment of the species-specific oligonucleotide composition “genome signature”.

The present study introduced the utility of BLSOM for characterizing sequence change of influenza virus genomes after invasion into human populations from other animal sources. An advantage of influenza virus genomes for bioinformatics studies is its high evolutionary rate, which allows for predictions obtained from all available data at a certain time to be checked within just a few years by using data newly accumulated after the first publication. This short time span is most suitable for testing the feasibility of a novel bioinformatics method and was the reason why the influenza viruses were chosen for the present study. Although all available sequences of influenza virus genomes were not a big data set at the time of the study, we introduced here a study of influenza viruses due to the above reason. This study could be conducted with high performance PCs rather than supercomputers and showed the wide applicability of BLSOM to genome studies of pathogenic microbes including viruses.

Influenza viruses present a significant threat to public health, as highlighted by the recent introduction of the swine-derived pandemic H1N1/09 [9-11] into human populations. Influenza virus pandemics have been often initiated by the introduction of a virus from animal sources followed by adaptation among humans through human-to-human transmission. The prediction of genome sequence changes and the surveillance of potentially hazardous viral strains that might cause new pandemics in human populations are important issues for the molecular evolutionary study of viruses, particularly influenza viruses [12,13]. We previously analyzed influenza A viruses with oligonucleotide BLSOMs and found that the oligonucleotide composition of strains isolated from avians and humans clearly differed from each other (i.e. host-dependent oligonucleotide composition) and the composition of the new pandemic H1N1/09 was different from that of human seasonal flu strains [14]. Furthermore, directional changes of oligonucleotide composition in H1N1/09 strains toward seasonal human strains were observed even within the first pandemic year. By analyzing newly accumulated data, the present study not only confirmed the prediction previously proposed, but also developed new strategies for predicting directional sequence changes and for surveilling potentially hazardous strains that may cause new pandemics in human populations.

## Methods

### Influenza virus genome sequences and their oligonucleotide frequencies

A total of 100,160 segment sequences derived from 12,395 influenza A and B virus strains were obtained in March/2012 from the NCBI Influenza Virus Resource (http://www.ncbi.nlm.nih.gov/genomes/FLU/) [15]. We obtained genome sequences from three H7N9 strains isolated in China from EpiFlu™ DATABASE (http://platform.gisaid.org/); A/Shanghai/1/2013, A/Shanghai/2/2013 and A/Anhui/1/2013. We calculated mono-, di-, tri-, and tetranucleotide frequency in eight genome segments of influenza virus strains, and summed up the frequencies of eight segments for each strain in order to conduct a genome-level analysis.

### Batch-learning self-organizing map (BLSOM)

SOM is an unsupervised neural network algorithm that implements a characteristic non-linear projection from the high-dimensional space of input data onto a two-dimensional array of weight vectors [3,4]. We have modified the conventional SOM for genome informatics to make the learning process and resulting map independent of the order of data input, and established a BLSOM [5-7]. BLSOM learning for oligonucleotide composition was conducted as described previously [7,8]; the average number of sequence data per neuron was chosen as four. BLSOM program was obtained from UNTROD, Inc. (y_wada@nagahama-i-bio.ac.jp).

## Results

### Mononucleotide BLSOM for all influenza A and B virus genomes

Viruses, including influenza viruses, are inevitably dependent on many host factors for their growth (e.g. pools of nucleotides, amino acids and tRNAs), and have to escape from antiviral host mechanisms such as antibodies, cytotoxic T cells, interferons, and RNA interference [16-18]. Thus, host-dependent differences in viral genomic sequences between strains isolated from different host species are to be expected. Influenza virus pandemics in human populations are often initiated by viral invasion from animal hosts and successive adaptation among humans through human-to-human transmission, as recently observed for the pandemic H1N1 strains (H1N1/09). Our previous BLSOM study [14] for di, tri and tetranucleotide compositions in genome sequences of 7,439 influenza A strains, which corresponded to all available data at that time, found clear separation (self-organization) of their sequences according to host, and features of the host-dependent oligonucleotide composition responsible for host-dependent separation were summarized by the following three rules. i) G- and C-rich oligonucleotides were more favored in avian strains than in human strains; G+C% effect. This G+C% effect was previously reported by Rabadan et al. [19]. ii) Oligonucleotides containing AG, CG or GA dinucleotide were more favored in avian strains than in human strains. This finding about the CG dinucleotide was consistent with the finding of Greenbaum et al. [20]. iii) Various characteristic cases, especially for tetranucleotides, could not be explained by the first two rules. For example, GGGG, a tetranucleotide composed only of G, was preferred mainly in human strains, while UCUU, a tetranucleotide rich in U, was preferred mainly in avian strains. The first G+C% rule should apparently be important for predicting directional changes of viral sequences, but the second and third rules should be important not only for predicting the directional sequence changes but also for potentially clarifying molecular evolutionary mechanisms of viral adaptation to hosts.

**Figure 1:**
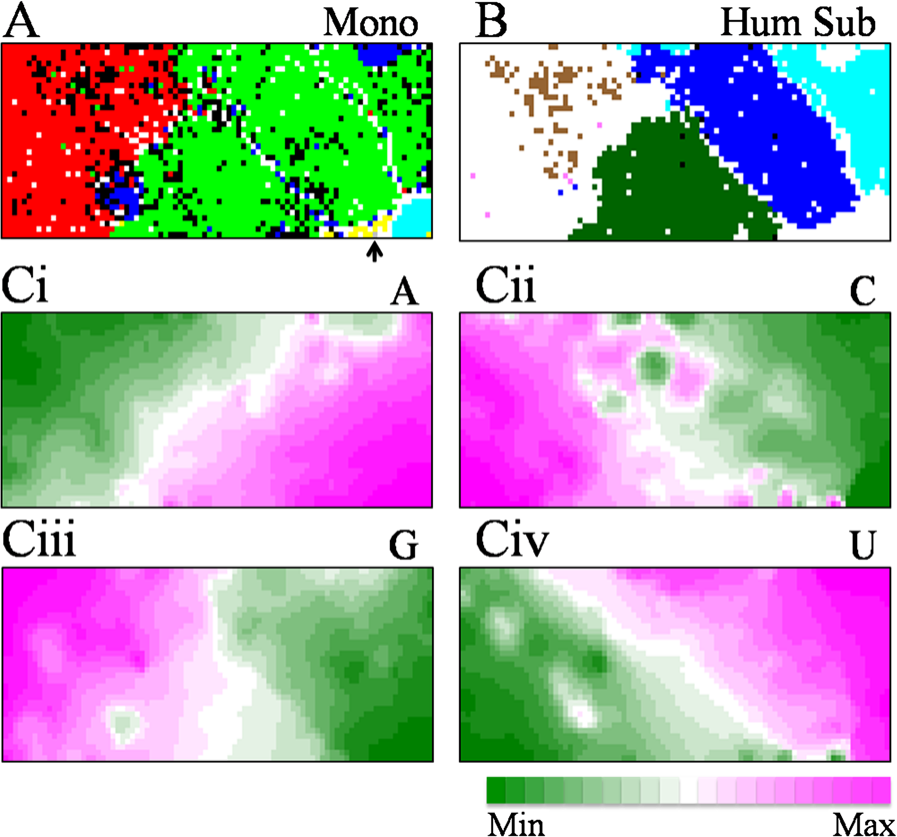
**Mononucleotide BLSOM for influenza A and B genome sequences. (A)** Mono. Mono-BLSOM was constructed for 12,067 influenza A and 328 B strains. Lattice points containing sequences from strains isolated from more than one host were indicated in black, and those containing sequences from one host were indicated in a color representing the host: avian (3,752 strains) colored in red, human (7,434 strains) in green, swine (783 strains) in blue, equine (95 strains), bat (3 strains) in grey, and influenza B virus (328 strains) in light blue. **(B)** Hum Sub. On the Mono-BLSOM, strains of each human subtype were specified in a color representing the subtype (H1N1 colored in light blue, H1N1/09 in dark green, H2N2 in geen, H3N2 in blue, H5N1 in brown, H7 in pink, H9 in pink, and others in green), and strains isolated from other animal hosts were achromatic. **(Ci-iv)** Occurrences of each mononucleotide at individual lattice points were sorted and classified into 21 groups and differing levels were indicated with different colors presented below the figure: wine red (high), dark green (low) and achromatic (intermediate).

In the present study, we newly introduced a BLSOM for mononucleotide composition (Mono-BLSOM) and analyzed 12,067 influenza A strains (isolated from avians, bats, equines, humans, and swines) plus 328 Influenza B virus strains (isolated from humans), which included ca. 5000 strains reported after our previous publication [14] (Figure 1A); in the case of H1N1/09 strains, approximately 80% of the sequences were newly reported after our previous publication. Although both influenza A and B virus genomes are composed of eight segments, we analyzed genomes of individual strains by summing up mononucleotide occurrences of eight segments for each strain in order to conduct a genome-level analysis. This enabled us to investigate sequence characteristics in each genome that are independent of functions of individual genes. The direct target of natural selection is a virion containing a full set of the eight segments, and this genome-level analysis should provide valuable and novel information for characterizing individual strains. Influenza virus possesses the negative-sense single-stranded RNA genome, and in the International DNA Sequence Databanks (DDBJ/EMBL/NCBI), sequences corresponding to the coding strand are registered and thus were analyzed in this study. When we consider the RNA genome itself, we have to make the exchange between A and U and between C and G.

In Figure 1A, lattice points containing only strains isolated from one host were indicated in a color representing the host and those including strains isolated from plural hosts were in black. Without information concerning the host during the BLSOM calculation, strains isolated from avian (red) or human (green) were primarily clustered (self-organized) according to host, forming large continuous colored territories. Swine strains (blue) formed two territories but equine strains (yellow) formed one compact territory. Influenza B strains (light blue) formed one territory located near the human and equine Influenza A territories and thus far away from the avian territory. This finding is consistent with the view that B strains have adapted well to human hosts through repeated epidemics in human populations exclusively. Bat strains (arrowed in Figure 1A) were also located far away from the avian territory and within mammal territories.

In Figure 1B, to visualize locations for human virus subtypes in separate, lattice points containing human strains belonging to one subtype were specified in one color representing the subtype and those including strains of plural subtypes were specified in black. Human seasonal H1N1, H3N2 and H1N1/09 were clearly separated from each other, forming their own territories; H1N1/09 strains were surrounded by seasonal human, avian (achromatic in Figure 1B and red in Figure 1A) and swine (achromatic in Figure 1B and blue in Figure 1A) strains. In contrast, human H5N1 strains (brown), which were introduced directly from birds but did not spread among humans, scattered within the avian territory, showing these H5N1 strains had characteristics of avian strains.

BLSOM provides a powerful ability to visualize occurrence levels of individual components (mononucleotides in this case) and thus supports an efficient knowledge discovery. Occurrences of each mononucleotide at individual lattice points were counted and sorted according to their level of occurrence, and the rank order was represented with a different color for each level of occurrence (Figure 1C); wine red (high occurrence) and dark green (low occurrence). G and C were richer on the left side of map where avian strains were mainly located, and therefore, a major portion of human seasonal strains was A- and U-richer than avian strains. This finding on Mono-BLSOM directly confirmed the G+C% rule (the host-dependent mononucleotide composition) found with the conventional oligonucleotide BLSOMs previously introduced [14].

Importantly, H1N1/09 strains (dark green in Figure 1B) had a mononucleotide composition roughly intermediate between avian and seasonal human strains. To be more exact, in a major portion of H1N1/09 strains, U occurrence (Figure 1Civ) was similar to that of avian strains, but A occurrence (Ci) was similar to that of human seasonal strains; G and C occurrences (Ciii and Cii) differed from those of avian strains in approximately half and only in a partial portion of H1N1/09 strains, respectively. This difference among mononucleotides could be effectively clarified by the newly introduced Mono-BLSOM and will be explained later in connection with the differential speed of directional changes found in human strains after introduction from other animal hosts.

### BLSOM for oligonucleotide composition normalized with mononucleotide composition

**Figure 2:**
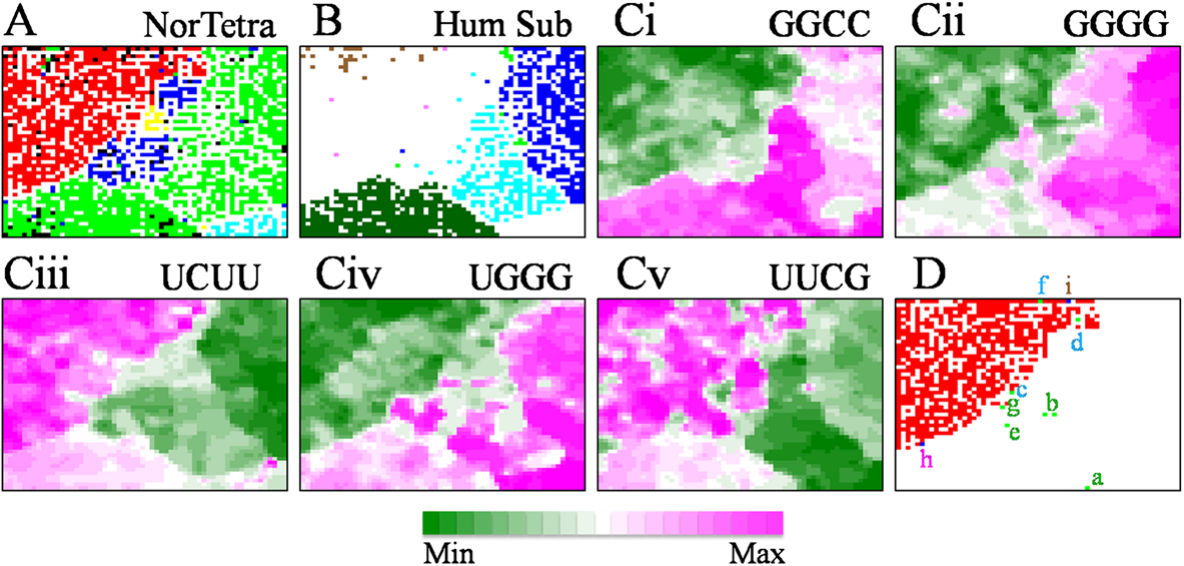
**Normalized Tetranucleotide BLSOM for all influenza A and B virus genome sequences.(A)** NorTetra. Lattice points on NorTetra-BLSOM were colored as described in Figure 1A. Green dots and many black dots in the avian territory primarily corresponded to H5N1 strains isolated from humans. **(B)** Hum Sub. On the NorTetra-BLSOM, lattice points containing different human subtypes were differentially colored as described in Figure 1B. **(Ci-v)** Normalized occurrences of each tetranucleotide at individual lattice points were sorted and indicated, as described in Figure 1C. **(D)** Map locations of avian strains of special interests and human H7N9 strains. Avian strains listed in Table 1 were indicated by green dots and by a-f colored as presented in Table 1, the avian H5N1 strain isolated from turkey in Virginia was indicated by a blue dot specified by pink-colored h, and three human H7N9 strains recently isolated were indicated by blue dots specified by brown-colored i.

Mono-BLSOM shows that mononucleotide composition is an important factor for determining host-dependent sequence characteristics in influenza genomes. We next studied the host-dependent oligonucleotide composition previously found with oligonucleotide BLSOMs [14], from a new and specific viewpoint that the host-dependent oligonucleotide composition would provide novel information concerning molecular mechanisms of viral adaptation to host; e.g. because certain oligonucleotides provide binding sites to proteins, the host-dependent oligonucleotide composition may reflect in part the difference in binding sites used for binding to host proteins. Oligonucleotide compositions, however, are inevitably affected by mononucleotide composition, and therefore, separation (self-organization) accomplished on oligonucleotide BLSOMs should be significantly affected by the host-dependent mononucleotide composition. In the present study, we newly developed an oligonucleotide BLSOM that was less affected by differences in mononucleotide composition between sequences. To be more precise, for each sequence, we first calculated the occurrence of each oligonucleotide expected from the mononucleotide composition of the sequence and the actual occurrence of this oligonucleotide in the respective sequence was divided by its expected value. BLSOMs for these normalized di-, tri- and tetranucleotide compositions were designated as NorDi-, NorTri- and NorTetra-BLSOM. NorTetra- and NorTri-BLSOMs were presented in Figure 2A and Additional file 1. On all BLSOMs, clear host-dependent separation was observed, and the number of black lattice points on these BLSOMs was far fewer than that on Mono-BLSOM, showing their high power of clustering according to host.

NorTetra-BLSOM showed that human and avian strains formed large territories clearly separated from each other; swine strains (blue) formed two territories but equine strains (yellow) formed one compact territory (Figure 2A). It was also clear in Figure 2B that human subtypes formed their own territories (light blue for seasonal H1N1, blue for H3N2 and dark green for H1N1/09). As observed on Mono-BLSOM, H1N1/09 strains were surrounded by avian, swine and seasonal human strains; human H5N1 strains (brown dots in Figure 2B and black or green dots in the avian territory in Figure 2A) were scattered within the avian territory.

In Figure 2C, the occurrence level of each tetranucleotide at each lattice point was shown with each level in the color, as described for Mono-BLSOM. Five examples of diagnostic tetranucleotides prominently contributing to the host-dependent clustering were presented in Figure 2C, and another six examples were presented in Additional file 2. In contrast to the separation on Mono-BLSOM, transitions between the high and low ranks (wine red and dark green, respectively) on NorTetra-BLSOM often coincided exactly with host territory borders, showing NorTetra-BLSOM’s high power of separation according to host. It also showed the tetranucleotides in charge of the separation, which could not be explained by the host-dependent mononucleotide composition. In detail, while avian strains were G- and C-richer than human strains, GGCC, GGGG and UGGG (G-rich tetranucleotides) were more favored by human seasonal influenza A and B strains than avian strains (Figure 2C). Among these three tetranucleotides, GGCC and UGGG were also favored by H1N1/09 strains, but the occurrence of GGGG was rather intermediate between avian and seasonal human levels. UCUU and UUCG were more favored by avian and H1N1/09 strains than seasonal human strains. These findings should provide predictive information about sequence changes in H1N1/09 strains and information concerning mechanisms for viral adaptation to human hosts.

### Retrospective time-series changes of human strains visualized on BLSOMs

**Figure 3:**
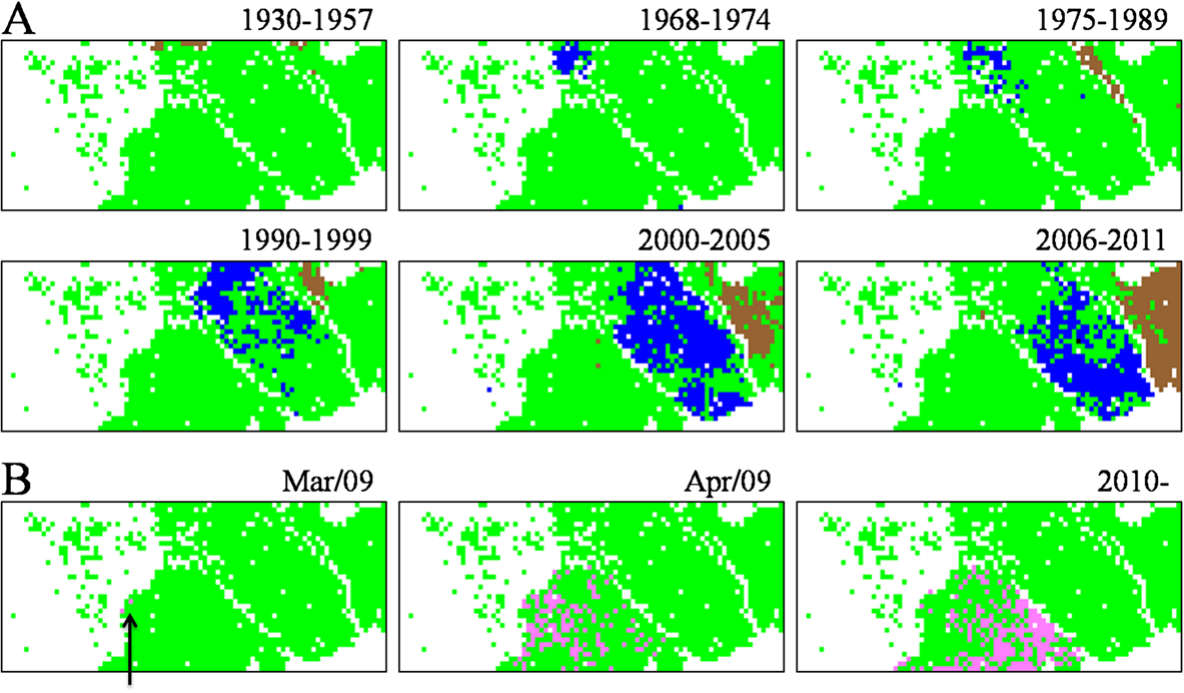
**Retrospective time**-**series changes observed for human seasonal and H1N1**/**09 strains on Mono**-**BLSOM presented in Figure**1**A. (A)** Human seasonal H1N1 and H3N2 strains isolated in the specified time period were indicated in the color representing the subtype (H1N1 and H3N2 colored in brown and blue, respectively), other human strains were left in green, and those from other hosts were achromatic. **(B)** H1N1/09 strains isolated in the specified time period were indicated in pink, and other human strains were left in green; and those from other hosts were achromatic. H1N1/09 strains isolated at a very early stage (March/09) were arrowed.

The prediction of genomic sequence changes in the near future is one important issue for the bioinformatics study of influenza viruses. Invader viruses will change their genome sequences on balance between stochastic processes of mutation and selective forces derived from various constraints, including those from a new host. Therefore, a certain level of changes may be predictable, at least in regard to specific aspects. To clarify actual directional changes, we next visualized retrospective time-series changes of human seasonal H1N1 and H3N2 strains on Mono- and NorTetra-BLSOM (Figure 3A and Additional file 3). Human seasonal H1N1 and H3N2 strains isolated in a specific time period were indicated in brown and blue, respectively; other human strains were left in green and strains isolated from other hosts were left achromatic. Seasonal human strains isolated in a very early stage (‘1930-1957’ for H1N1 and ‘1968-1974’ for H3N2) were located near the avian territory (achromatic in Figure 3A and red in Figure 1A) and pandemic descendants isolated in later periods moved apart from the avian territory, showing time-series directional changes. The directional changes were also observed on NorTetra-BLSOM (Additional file 3).

Figure 3B similarly visualized time-series changes of H1N1/09 strains on Mono-BLSOM; strains isolated in a specific time period were indicated in pink. Strains isolated in March/09 (arrowed) and a major portion of the strains isolated in April/09 were located in the vicinity of avian and swine territories, but those isolated after 2009 were primarily located near the human seasonal flu territory and thus apart from the avian territory. Among the H1N1/09 strains isolated after 2009, approximately 80% were isolated after our previous publication, and therefore, directional sequence changes previously predicted for H1N1/09 strains were confirmed by utilizing the newly accumulated data.

**Figure 4:**
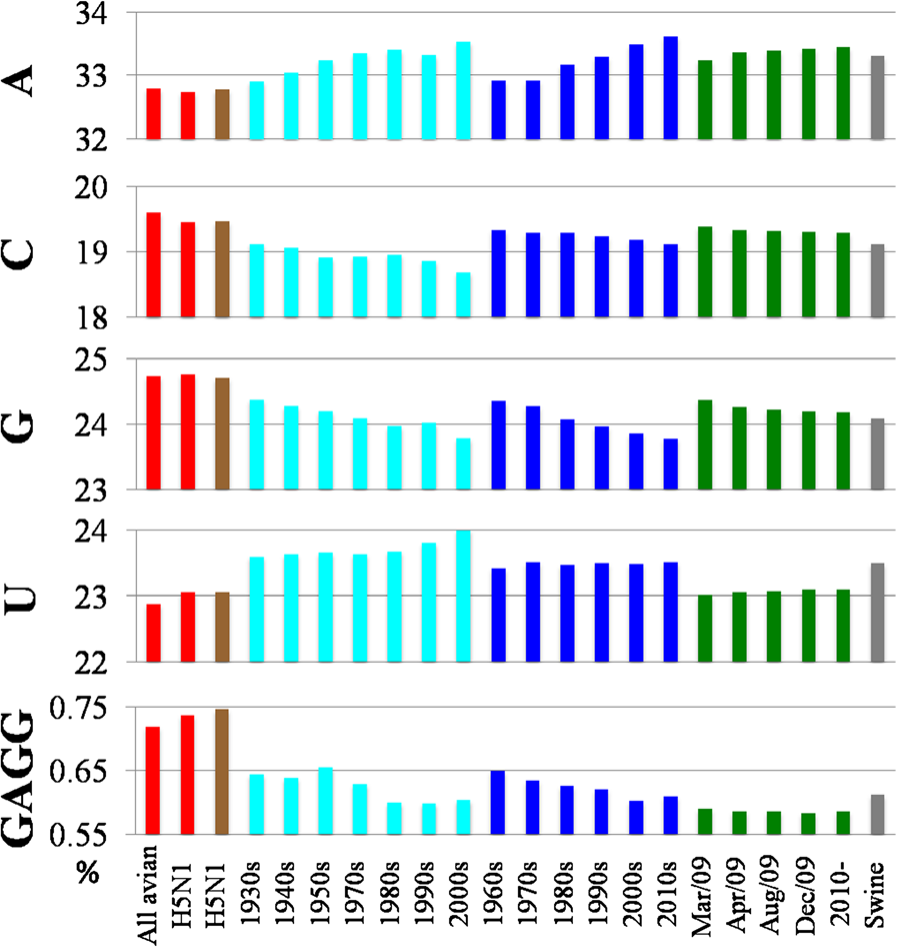
**Time**-**series changes of mono**- **and tetranucleotide frequencies (%).** The average frequency of each mononucleotide and of one tetranucleotide GAGG among strains belonging to one human subtype isolated in a specific time period was shown with a colored bar specifying the subtype (H1N1 and H3N2 colored in light blue and blue, respectively). In the case of avian (red) and swine (grey) and of human H5N1 (brown) strains, the average frequency of each mononucleotide was calculated for all strains independently of isolated years.

Time-series change of mononucleotide composition observed on Mono-BLSOM can be verified even by analyzing the average composition of human strains isolated in different time periods (Figure 4). The average of A or U occurrence for all avian strains (red), as well as avian H5N1 strains (red), was lower than that for human subtypes isolated in all time periods, except for human H5N1 strains (brown); and the opposite was observed for G and C. Furthermore, A and U occurrences increased over time while C and G occurrences decreased, confirming the time-series directional changes found on Mono-BLSOM. Importantly, the rapidity of the directional change appeared to differ among mononucleotides; A and G > C > U. Time-series changes of A and G were clearly observed for both H1N1 and H3N2, but that of U was less clear, especially for H3N2, indicting a slow rate of the change. The reason why the U change in H3N2 was less clear than in H1N1 should be the shorter time span analyzable for H3N2. The C change appeared to be intermediate between the above two groups.

Although time-series changes in H1N1/09 analyzable for three years were less clear than those in seasonal subtypes, the difference among mononucleotides aforementioned for seasonal subtypes was supported (dark green bars in Figure 4); the change of U, for which the lowest rate was predicted, was merely detected, but for other mononucleotides, changes at a low level but in the same direction to that in seasonal subtypes were observed.

Time-series changes of tetranucleotide compositions were similarly analyzed, and an example of tetranucleotides (GAGG) was presented in Figure 4. Clear time-series changes were observed for seasonal subtypes, and occurrence for H1N1/09, but not human H5N1 (brown), clearly differed from those of avian strains but was similar to those of seasonal human subtypes.

### A strategy for finding potentially hazardous strains

Another important issue for bioinformatics studies of influenza viruses is a search for strains that will become hazardous in the near future. In our previous paper [14], we proposed that the avian and swine strains located in the close vicinity of human territories on oligonucleotide BLSOMs were hazardous strain candidates, because these strains had oligonucleotide compositions with a higher similarity to human strains than those located away from human strains. To extend this approach and develop an additional strategy for surveillance of non-human strains with a high risk potential, we assume here that the seasonal human H1N1, H3N2 and H1N1/09 strains isolated at a very early stage may have characteristics that potentially prepare them for efficient human-human transmission. We thus focused on specific tetranucleotides, whose occurrences in these specified human strains were distinct from those in most avian strains; refer to GAGG listed in Figure 4, as an example. In practice, we first calculated the average occurrence of each tetranucleotide in human H1N1 and H3N2 strains isolated at a very early stage defined in Figure 3 and in H1N1/09 strains isolated in 2009; we then selected the tetranucleotides, for which each of the above-mentioned three averages was higher or lower than occurrences of more than 80% of avian strains; a similar result was obtained when more than 85% was chosen (our unpublished data). This selection was based on the assumption that a limited portion of avian strains may have a human-type preference for some tetranucleotides, as explained below for avian strains with a high risk potentiality. 13 higher and 10 lower cases of the diagnostic tetranucleotides were thus selected and listed below Table 1. Using these high or low diagnostic tetranucleotides as assessment criteria, we then searched for avian strains with a significant level of a human-type preference. In practice, for each avian strain, we counted the high or low diagnostic tetranucleotides whose occurrences were judged as human-type, by referring to each of the three averages for human subtypes isolated in the very early stage. Table 1 listed avian strains with an apparent human-type preference and Figure 2D showed their locations on NorTetra-BLSOM with alphabetical letters noted in Table 1. Two H1N1 strains isolated from turkey in Ontario in 2009 had human-type preferences for 18 and 17 tetranucleotides out of a total of 23 diagnostic tetranucleotides; designated here as score 18 and 17 points. These avian strains were shown to be human-to-bird transmitted H1N1/09 by the phylogenetic tree analysis [21] and actually were located within the H1N1/09 territory on NorTetra-BLSOM (Figure 2D). Two H1N1 strains isolated from turkeys in US scored 17 points, and were located within a swine territory near a border to the human territory, indicating swine-to-bird transmission. These findings supported suitability of the choice of the diagnostic tetranucleotides.

**Table 1:** Avian strains with high scores

**Point**	**Subtype**	**Year**	**Country**	**Strain name**	**For position**
*18*	*H1N1*	*2009*	*Canada*	*A/turkey/Ontario/FAV110/2009*	*a*
*17*	*H1N1*	*2009*	*Canada*	*A/turkey/Ontario/FAV114-17/2009*	*a*
*17*	*H1N1*	*1992*	*USA*	*A/turkey/IA/21089-3/1992*	*b*
*17*	*H1N1*	*1988*	*USA*	*A/turkey/NC/17026/1988*	*b*
**15**	**H4N2**	**2006**	**USA**	**A/pekin duck/California/P30/2006**	**c**
**11**	**H4N8**	**2006**	**Japan**	**A/slaty-backed gull/Japan/6KS0191/2006**	**d**
**11**	**H4N8**	**2006**	**Japan**	**A/rufous-necked stint/Japan/6KS0279/2006**	**d**
**11**	**H4N8**	**2006**	**Japan**	**A/rufous-necked stint/Japan/6KS0242/2006**	**d**
**11**	**H3N8**	**2007**	**USA**	**A/cinnamon teal/California/44287-325/2007**	**c**
*11*	*H1N2*	*2001*	*USA*	*A/duck/NC/91347/01*	*e*
**10**	**H6N2**	**2002**	**China**	**A/wild duck/Shantou/867/2002**	**f**
**10**	**H4N8**	**2006**	**Japan**	**A/slaty-backed gull/Japan/6KS0185/2006**	**d**
*10*	*H3N2*	*2004*	*USA*	*A/turkey/Illinois/2004*	*g*

High diagnostic tetranucleotides: AAGU, ACUU, AUCA, AUGA, AUUA, AUUU, CUUU, GGCC, UCAU, UUAA, UUAU, UUCA, and UUUG.

Low diagnostic tetranucleotdes: ACGC, AUUG, CUCC, CUGA, GAGC, GAGG, GCAG, GGAC, UCUU, and UGUG.

The avian strains suspected to be directly transferred from humans or swines were indicated in italic. Other strains were indicated in bold.

**Figure 5:**
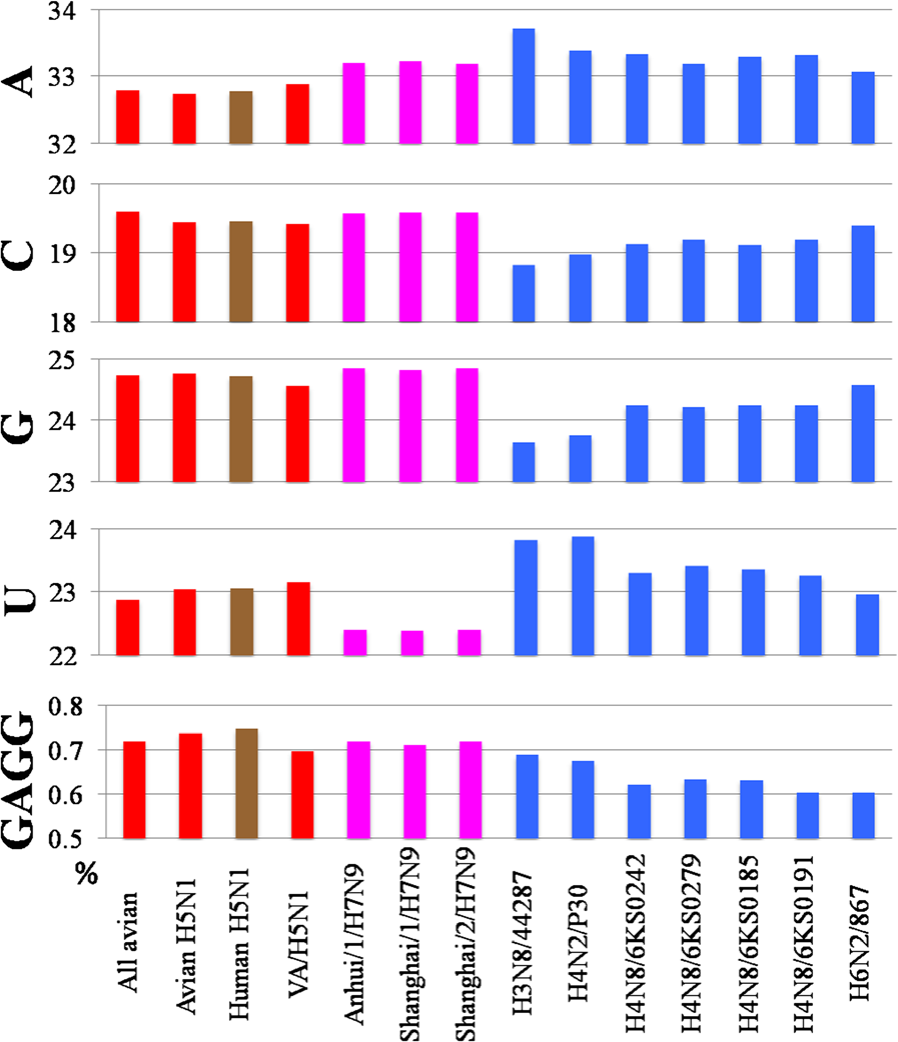
**Mono**- **and tetranucleotide frequencies (%). **The average frequency of each mononucleotide and of GAGG for all avian and avian H5N1 strains (red) and for human H5N1 strains (brown) was shown with a colored bar. The frequency for the avian H5N1 strain isolated from turkey in Virginia (VA/H5N1, red), the avian H3, H4 and H6 strains listed in Table 1 (blue) and three human H7N9 strains (pink) also was shown with a colored bar.

Importantly, an H4N2 strain isolated from Pekin duck in California also had a very high score equivalent to the above-mentioned avian strains transmitted from human or swine, though the H4N2 subtype has not caused epidemics among humans. When avian strains with characteristics similar to the Pekin duck strain will invade to humans, this may cause human-to-human transmission with a significant probability: i.e. these candidate strains may have high risk potential. H4N8, H3N8 and H6N2 strains isolated from various birds in various places listed in Table 1 also had relatively high scores, although these subtypes have also not caused epidemics in human populations. We next analyzed the mononucleotide compositions for these strains with the high scores (Figure 5). The occurrence of A and U was clearly higher than the average level of avian stains and similar to those of seasonal human H1N1, H3N2 and H1N1/09 strains, supporting a view that these strains may have the high risk potential.

In contrast, all known human H5N1 strains, which had not caused epidemics in human populations, had low scores (≤ 5), and therefore, were not listed in Table 1. An avian H5N1 strain isolated from turkeys in Virginia had a higher score (6 points) than all known human H5N1 strains, indicating this avian H5N1 strain may have a higher possibility of human-human transmission than the known human H5N1 strains. The mononucleotide composition in this strain had a clearly lower level of the human-type preference than that of the aforementioned H4N2, H4N8, H3N8 and H6N2 strains but a higher level than that of human H5N1 strains (Figure 5).

During revision process of this manuscript, transmission of H7N9 strains from birds to humans was reported. Genome sequences from three H7N9 strains isolated in China were obtained from EpiFlu™ DATABASE. Their scores (4 points) were higher than the average of known human H5N1 strains (2.1) and equivalent to the second highest level of the human H5N1 strains. Mononucleotide composition in these H7N9 strains had a clearly lower level of the human-type preference than that of the aforementioned H4N2, H4N8, H3N8 and H6N2 strains (Figure 5). The A (but not U) occurrence of these H7N9 strains was higher than that of the average of avian strains; C and G occurrence was similar to the average of avian strains. Taking these findings into account, we think that the risk level of epidemics of these H7N9 strains in the human population may not be evidently high.

On NorTetra-BLSOM in Figure 2D, the strains suspected to be directly transferred from humans or swines (shown in italic in Table 1) were located within human or swine territory (a, b, e, and g in Figure 2D), but others (shown in bold in Table 1) were located in the avian territory but mainly near borders between avian and human/swine territories (c, d and f in Figure 2D). The avian H5N1 strain isolated in Virginia also was in the border between avian and human territories (h in Figure 2D). The finding that there were avian H5N1 strains with a higher level of human-type preference than all known human H5N1 strains supports a view that avian H5N1 strains will acquire a much higher level of human-type preference. Three human H7N9 strains were located in the avian territory but near the border to the human territory on NorTetra-BLSOM (i in Figure 2D), where avian H9N2 strains isolated mainly in China were located. This finding indicated the evolutionary origin of these H7N9 strains.

Because strains listed in Table 1 were selected on the basis of a criterion independent of BLSOM, this finding supported our previous proposal that non-human strains located in the close vicinity of human territories on BLSOMs may be candidates for potentially hazardous strains. By combining mutually independent bioinformatics methods, we can develop a strategy for efficient and large-scale surveillance of potentially hazardous strains that may cause new pandemics in human populations in the near future.

## Discussion

We first discussed characteristics of the present alignment-free clustering method “oligonucleotide BLSOM”, by comparing it with the phylogenetic tree analysis. Undoubtedly, the phylogenetic tree analysis has provided the most powerful strategy to study evolutionary processes of gene and protein sequences [10-13,22,23]. It should also be stressed here that the diversity of analytical methods, especially those based on different principles, is important for unveiling a wide range of characteristics hidden in genome sequences. Oligonucleotide composition is least affected by functions of individual genes, and therefore, the genome-level analysis can be easily conduced and the hidden characteristics in the genome that are not directly related to the gene function can be unveiled. Usefulness of this type of sequence alignment-free analysis has been proven by evolutionary studies of codon usage [24]. The direct target of natural selection is a virion itself, and in the present study, we analyzed oligonucleotide compositions for a total of eight segments in order to characterize individual virus strains at a genome-level.

It should also be noted that, at the onset of a new pandemic, reassortment of virus genome segments in a certain host (e.g., swine) and successive invasion of the new reassortant into human populations were often observed [25,26]. Therefore, separate analyses of eight segments also are undoubtedly important. In our previous study [14], oligonucleotide compositions of eight segments also were separately analyzed, and this showed clear host-dependent clustering of each segment on oligonucleotide BLSOMs, even thought the length of the shortest segment (segment 8) is approximately 0.8 kb. The results obtained with the gene-level BLSOM analysis were primary consistent with those obtained with the phylogenetic tree analyses [14].

The strategies developed in this paper may not be the final form of actual surveillance at the present moment. For example, diagnostic tetranucleotides listed below Table 1 were selected by the criterion that their occurrences should satisfy every requirement observed for the three human subtypes. This criterion appears to be suitable for searching for strains with a very high risk potential, but may be too strict for actual surveillance. The main point of our proposal is that an integration of studies of oligonucleotide compositions (e.g. tri-, tetra- and pentanucleotides), including studies with oligonucleotide BLSOMs, can provide a conducive surveillance strategy, because the host-dependent oligonucleotide composition that cannot be explained by the host-dependent mononucleotide composition should relate, at least in part, to host-adaptation mechanisms of the virus. We recently developed BLSOMs for peptide compositions [27] and found clear host-dependent clustering of influenza virus proteins on peptide BLSOMs (our unpublished data). Because peptide composition is more directly related to gene function than oligonucleotide composition, analyses of peptide compositions should provide strategies for prediction of sequence changes and surveillance of potentially hazardous strains from a new and distinct viewpoint. Continuous monitoring of all sequenced influenza virus strains by oligonucleotide- and peptide-BLSOMs may provide a model example for world-wide surveillance of potentially hazardous zoonotic viral strains, for which big sequence data will become available in the near future.

## Conclusions

BLSOM can simultaneously characterize millions of sequences from infectious microbes and viruses, which have become available because of their medical importance. BLSOM’s powerful visualization on one plain enables us to efficiently obtain profound knowledge from big data by encompassing the data. We can use BLSOM for study of big sequence data obtained from any genomes and have successfully applied oligonucleotide BLSOMs to phylogenetic classification of millions of metagenomic sequences obtained from various environmental samples [8].

## Competing interests

The authors declared that there are no conflicts of interests.

## Authors’ contributions

YI conceived the approach and conducted this analysis. TA developed the algorithm. YW carried out modifications of the algorithm. KW developed, implemented and validated the algorithm. TI supervised the program development and this study. All authors read and approved the final manuscript.

## Pre-publication history

The pre-publication history for this paper can be accessed here:

http://www.biomedcentral.com/1471-2334/13/386/prepub

## Supplementary Material

Additional file 1 NorTri-BLSOMs for influenza A and B virus genome sequences. Click here for file

Additional file 2 **NorTetra-BLSOMs for influenza A and B virus genome sequences.** Additional 6 examples of diagnostic tetranucleotides were presented. Click here for file

Additional file 3 Retrospective time-series changes for seasonal human and H1N1/09 strains on NorTetra-BLSOM. Click here for file
