# Supplementary figures and images for: Novel bioinformatics strategies for prediction of directional sequence changes in influenza virus genomes and for surveillance of potentially hazardous strains
Source: BMC Infect Dis. 2013 Aug 21;13:386. doi: 10.1186/1471-2334-13-386 (PMC3765179; doi:10.1186/1471-2334-13-386)

Additional file 1

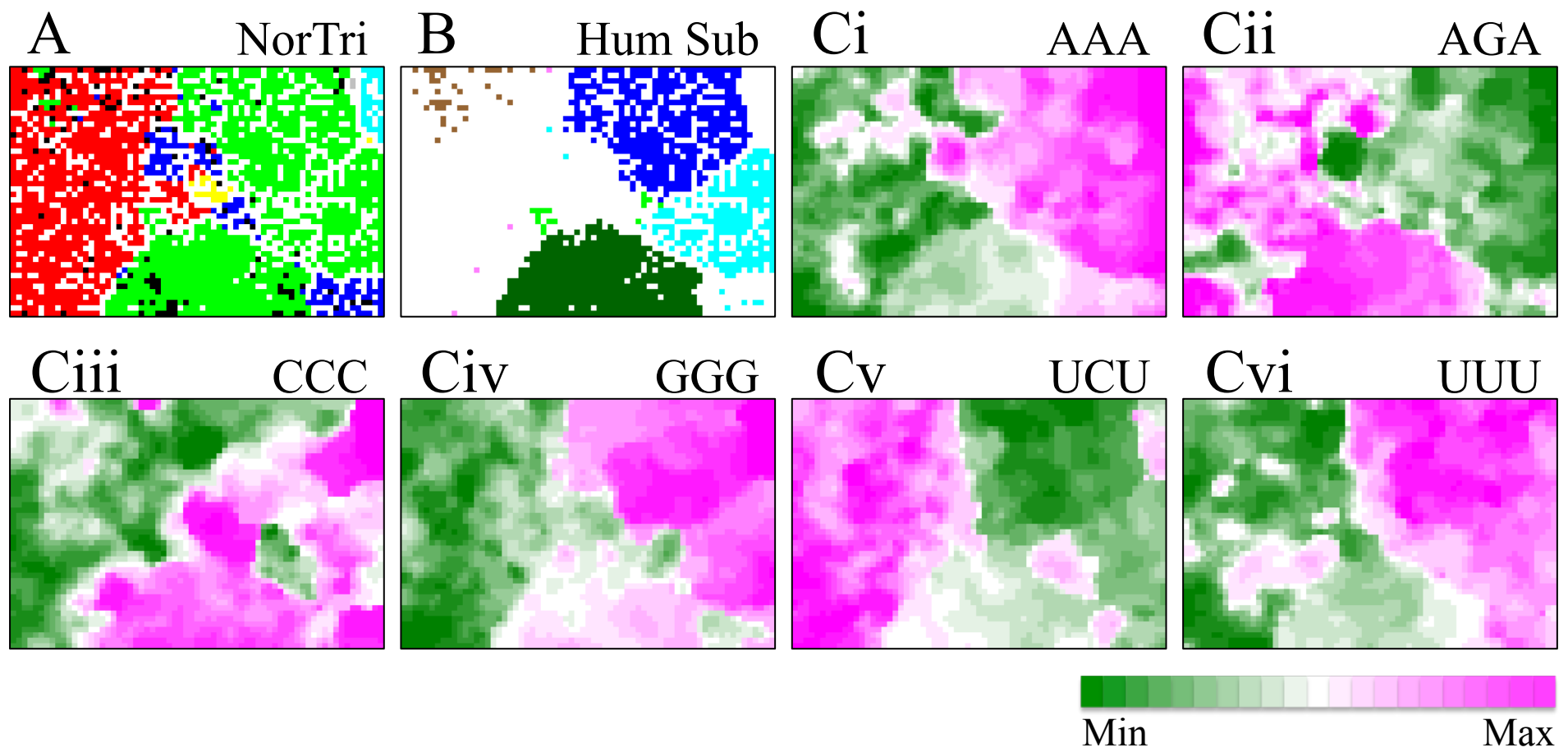

Supplement: Additional file 1 — NorTri-BLSOMs for influenza A and B virus genome sequences. [file 1471-2334-13-386-S1.pdf]

Additional file 2

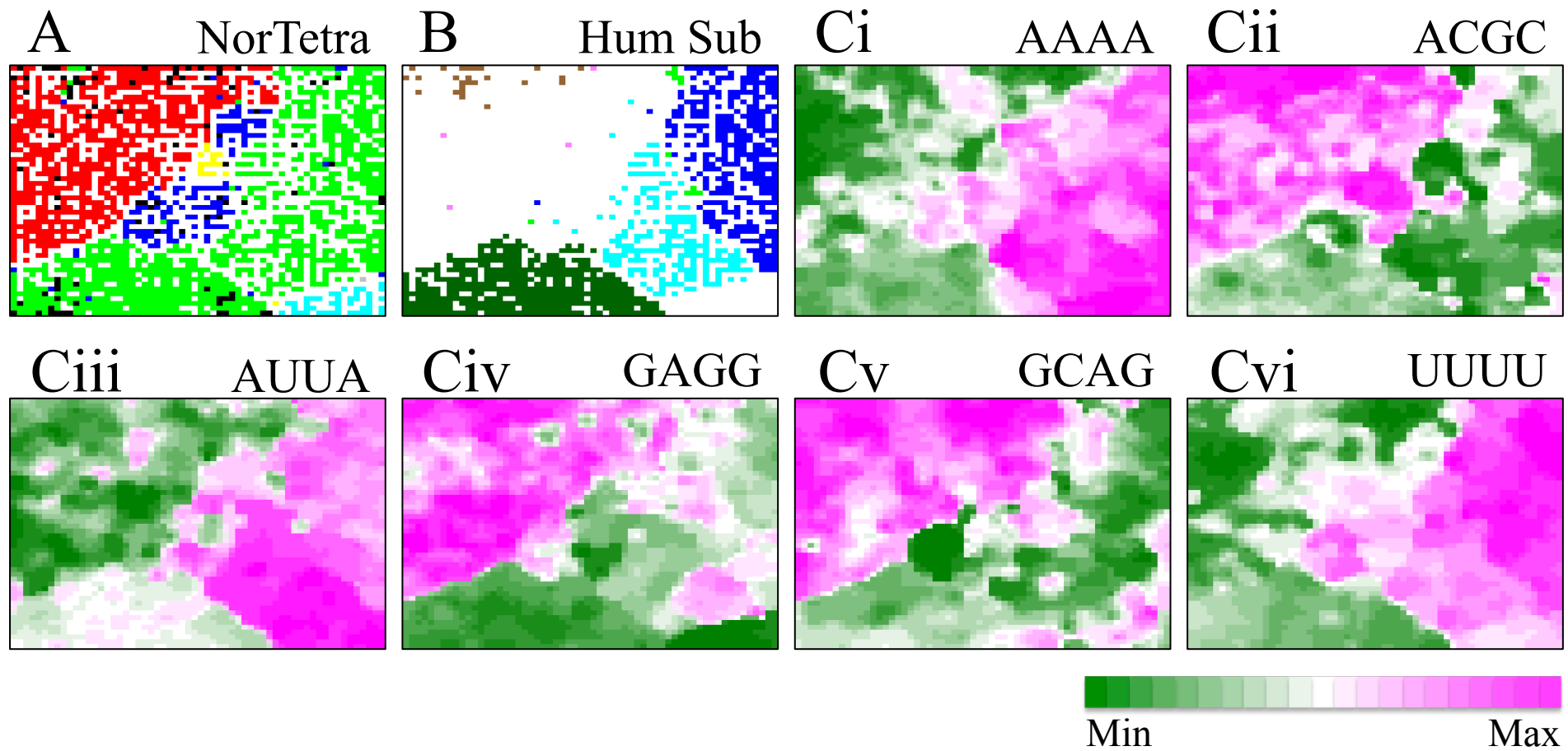

Supplement: Additional file 2 — NorTetra-BLSOMs for influenza A and B virus genome sequences. Additional 6 examples of diagnostic tetranucleotides were presented. [file 1471-2334-13-386-S2.pdf]

### Additional file 3

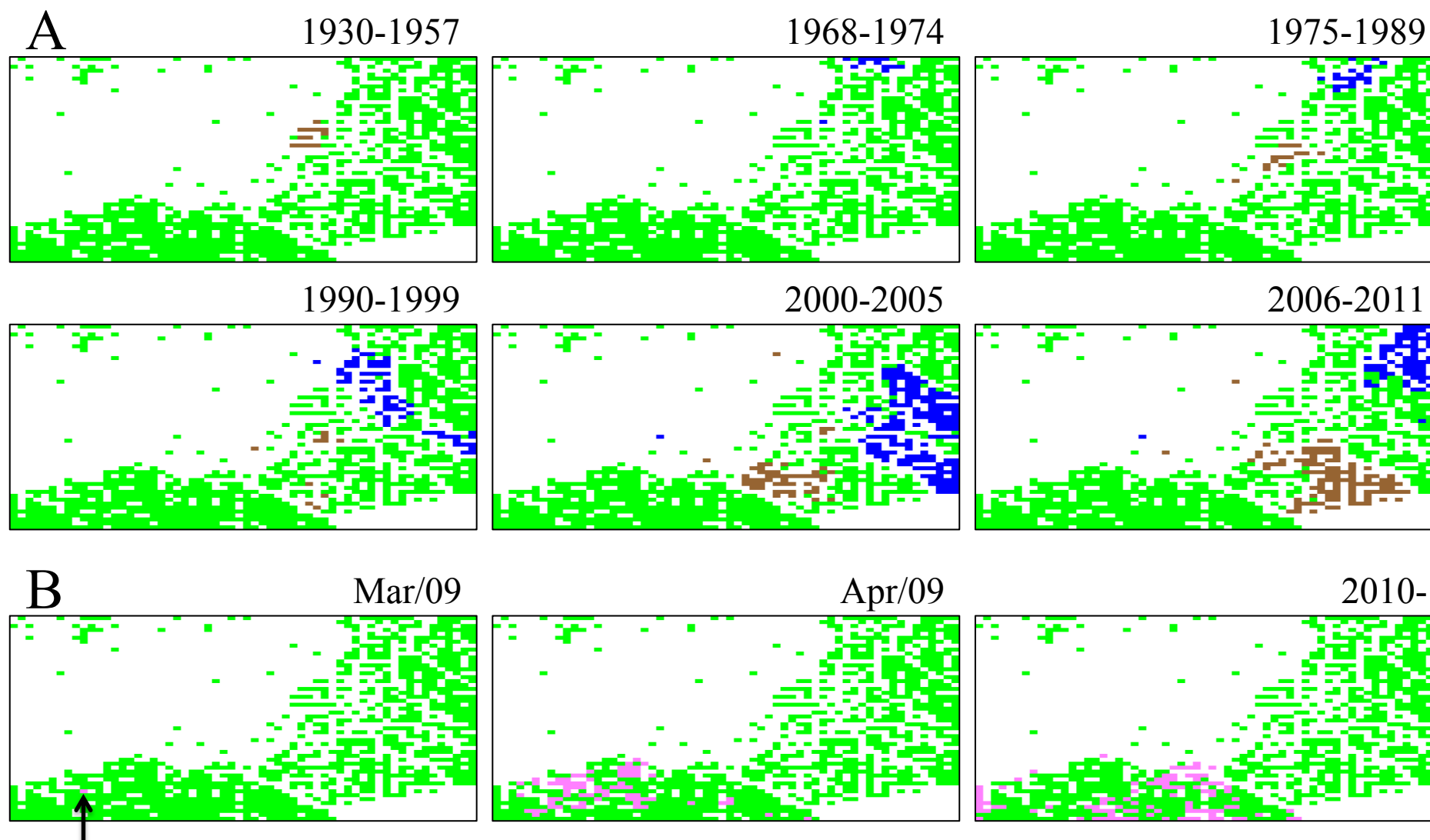

Supplement: Additional file 3 — Retrospective time-series changes for seasonal human and H1N1/09 strains on NorTetra-BLSOM. [file 1471-2334-13-386-S3.pdf]
